# Subtle right ventricular dysfunction in asymptomatic chronic heavy cigarette smokers: a speckle tracking case-control study

**DOI:** 10.1186/s43044-021-00151-y

**Published:** 2021-03-16

**Authors:** Mohammad Iqbal Janhangeer, Ghada Youssef, Weal El Naggar, Dalia El Remisy

**Affiliations:** Cardiology Department, Kasr Al Ainy Faculty of Medicine, Cairo University, Cairo, Egypt

**Keywords:** Strain, Right ventricle, 2D echocardiography

## Abstract

**Background:**

Chronic heavy cigarette smoking can affect the right ventriclular function. The standard echocardiography may not show early right ventricular functional changes, and a more sensitive measure is needed. The aim of this work was to evaluate the subtle subclinical effects of chronic heavy cigarette smoking on the right ventricular function. The study included 55 healthy asymptomatic chronic heavy cigarette smokers (smoking history of at least 5 pack-years and a daily cigarette consumption of at least 1 pack) and 35 healthy non-smoking control subjects. Patients underwent a full clinical assessment and a conventional as well as a 2D-speckle tracking transthoracic echocardiography of the right ventricle and data was compared between the 2 groups.

**Results:**

The mean age was 32.9 ± 7.2 years in smokers and 30.9 ± 7.9 years in non-smokers (*p* = 0.227). The 2 groups showed comparable conventional right ventricular systolic and diastolic functions. Smokers showed a significantly lower (less negative) right ventricular global longitudinal strain (− 19.0 ± 3.2% vs. − 24.5 ± 3.5%, *p* < 0.001). Patients with a higher daily cigarette consumption showed a poorer right ventricular global longitudinal strain (*p* = 0.014).

**Conclusion:**

Chronic heavy cigarette smoking can adversely affect the right ventricular function, a finding that can be easily missed by conventional echocardiography and can be better detected by the right ventricular speckle tracking.

## Background

More than one billion people, about one quarter of adults worldwide, smoke tobacco [1]. Tobacco is currently the greatest preventable cause of death in the world, killing up to half the people who smoke it, which is more than 7 million people worldwide annually [2]. Current data suggest that smoking is a public health problem in Egypt. Prevalence of cigarette smoking was 46.4% among males and 0.2% among females in the Global Adult Tobacco Survey (GATS) Egypt Country Report 2009 [1].

Cigarette smoking is one of the major risk factors for coronary heart disease, stroke, atherosclerosis, aortic aneurysm, peripheral vascular disease, and subclinical cerebrovascular disease [3]. Kaplan et al. reviewed the possible mechanisms by which cigarette smoke can directly affect the myocardium thereby causing smoking cardiomyopathy, and concluded that oxidative stress, inflammation, metabolic impairment, and cell death were the possible factors responsible for cardiac remodeling after chronic cigarette exposure [4].

The right ventricle (RV) has long been regarded as the forgotten side of the heart and little attention has been paid to its assessment [5]. Nowadays, however, there is no doubt that it plays a critical role in the prognosis of different cardiovascular diseases. The function of the RV is a strong determinant of the prognosis for patients with congestive heart failure, ischemic heart disease, cardiomyopathy, pulmonary arterial hypertension and congenital heart defects [6–12]. Therefore, there is a great need to evaluate its function accurately.

There are few studies that have focused on the effects of smoking on the right ventricular function, and they have shown conflicting results [13–15].

The present study aims to find out whether chronic cigarette smoking, in otherwise healthy individuals, can cause subtle dysfunction of the right ventricle by applying a 2D speckle tracking echocardiography (2D-STE) as well as other conventional echocardiographic methods of assessment of the right ventricular function.

## Methods

This is a cross-sectional, observational, case-control study which was conducted at the Cardiovascular Medical Department after approval from the faculty’s Ethical Committee. Ninety subjects volunteered to participate in the study of whom 55 were healthy, asymptomatic heavy chronic cigarette smokers aged 45 years or less and 35 were healthy never-smokers control subjects. Healthy current cigarette smokers should have had a smoking history of at least 5 pack-years and a daily cigarette consumption of at least 1 pack (20 cigarettes) per day. Exclusion criteria included atrial fibrillation and other arrhythmias, poor echocardiographic window, the presence of > 1 segment (out of the six segments of the RV) without clearly defined endocardial and epicardial borders, other substance abuse, obesity (BMI > 30 kg/m^2^), chronic obstructive pulmonary disease (COPD), elevated pulmonary artery systolic pressure (PASP) more than 35 mmHg, and greater than mild valvular lesions. After obtaining an informed verbal consent from all subjects, a detailed physical assessment was performed followed by a transthoracic echocardiography.

Subjects were asked to stop smoking for at least 30 min before undergoing echocardiography to minimize the effects of acute smoking on the heart. They were also requested not to consume any foods or drinks containing caffeine (e.g., coffee) for at least 3 h before the echocardiographic study.

Echocardiography was performed using the commercially available machine (PHILIPS, Epic 7), equipped with a 2.5-MHz phased array transducer. Analysis of the Speckle images was performed using the Q-lab10 software available both on the echocardiography machine as well as on a laptop.

All the standard echocardiography views were recorded in addition to the apical RV-focused view. The systolic and diastolic functions of the left ventricle were assessed according to the American Society of Echocardiography guidelines [16–18]. Conventional parameters of the right ventricular systolic and diastolic functions included tricupsid annular plane systolic excursion (TAPSE), tissue Doppler imaging (TDI) tricuspid valve S-wave velocity, and RV Tei Index calculated as the sum of the isovolumic contraction time (ICT) and the isovolumic relaxation time (IRT) divided by ejection time (ET) (calculated as follows: ICT + IRT/ET), and E^/^ and A^/^of the lateral tricuspid annulus, right ventricular fractional area change (FAC), right atrial volume, E and A pulse wave Doppler velocity over the tricuspid inflow, diameter and inspiratory collapsibility of the inferior vena cava (IVC), peak tricuspid regurgitant jet velocity, and estimated pulmonary artery systolic pressure (calculated as right ventricular systolic pressure + right atrial pressure).

Video clips of the RV-focused view were recorded, ensuring a clear definition of the RV endocardial and epicardial borders. A minimum of three beats were recorded. The depth, the sector width, and the frequency were adjusted to obtain a frame rate of at least 50 Hz. The operator was blind of the smoking history of the studied subjects and clips were analyzed later, using the offline speckle tracking Q-lab software to determine the global longitudinal strain (GLS) of the right ventricle.

**Fig. 1:**
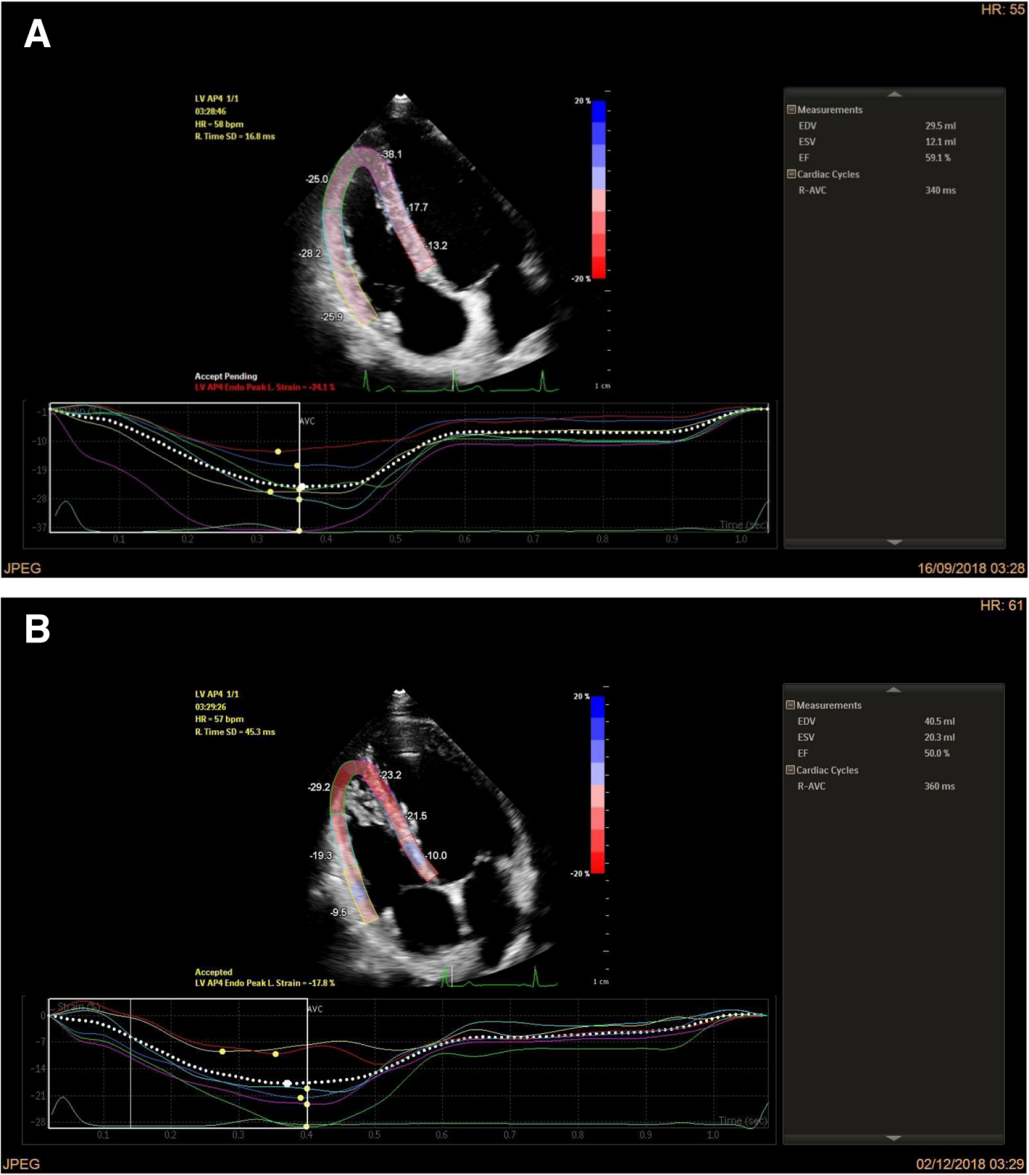
Strain of the six segments of the right ventricle and GLS of the right ventricle of one of the non-smoking control subjects (**a**) and one of the chronic cigarette smokers (**b**)

As the available machine did not include a software for RV speckle analysis, we used the left ventricular software and we manually adjusted the tracked images to fit the RV shape. It was assumed that the aortic valve closure time was not significantly different from the pulmonary valve closure time. The software automatically calculated the aortic valve closure time from the left ventricular apical three-chamber view. The region of interest (ROI) was manually selected and the software automatically traced the endocardial and epicardial borders of the RV. Necessary adjustments were made to ensure adequate tracking of the right ventricular free wall and the interventricular septum. Care was taken not to include the right atrial wall and the epicardium in the region of interest. The RV speckle tracking images included analysis of 6 segments; the basal, mid, and apical segments of the RV septal and free walls. Two examples of the strain of the 6 segments of the RV together with the RV GLS of both groups are shown in Fig. 1.

Finally, the software automatically calculated the Lagrangian strain of each of the 6 segments using the end-diastole as the reference point. The GLS of the RV was also automatically calculated. Pooled data suggest that global longitudinal RV free wall strain > − 20% (i.e., < 20% in absolute value) is likely abnormal [16].

### Statistical analysis

Categorical data was presented as numbers and percentages while continuous data was presented as mean and standard deviation (SD). Comparison of means between groups was performed using the independent student *t* test or one way ANOVA test, as convenient. Correlation between continuous variables was done using Pearson correlation test. All statistical analysis was done using an SPSS 26 program. A *p* value of < 0.05 was considered significant.

## Results

The demographic data of the 2 groups were comparable (see Table 1). While chronic heavy cigarette smokers had a higher pulse rate than non-smokers, the difference in the blood pressure between the two groups was not significant.

**Table 1:** Demographic data and clinical characteristics of the 2 groups

Variable	Smokers (mean ± SD)	Non-smokers (mean ± SD)	***p*** value
Age/year	32.9 ± 7.2	30.9 ± 7.9	0.227
BMI (kg/m^2^)	24.5 ± 3.1	25.0 ± 2.5	0.481
BSA/m^2^	1.90 ± 0.16	1.89 ± 0.19	0.902
SBP/mmHg	120 ± 9	117 ± 7	0.132
DBP/mmHg	77 ± 7	75 ± 7	0.359
Pulse (beats/min)	75 ± 9	69 ± 8	0.001

*BMI* body mass index, *BSA* body surface area, *DBP* diastolic blood pressure, *SBP* systolic blood pressure

Both smokers and non-smokers had normal LV systolic and diastolic functions. The conventional methods for the assessment of the systolic and diastolic functions of the RV were normal and comparable for both groups as shown in Table 2. Chronic heavy cigarette smokers were found to have a statistically significant higher tricuspid valve (TV) a′ velocity and a lower TV e′/a′ ratio as compared to non-smokers.

**Table 2:** Conventional echocardiographic parameters of the systolic and diastolic RV functions of both groups

Variable	Smokers (mean ± SD)	Non-smokers (mean ± SD)	***p*** value
RV basal diameter, cm	3.5 ± 0.4	3.4 ± 0.5	0.070
RV mid cavity diameter, cm	3.3 ± 0.4	3.2 ± 0.4	0.322
RV longitudinal diameter, cm	7.1 ± 0.6	7.0 ± 0.5	0.603
TAPSE, cm	2.3 ± 0.4	2.2 ± 0.3	0.271
RV S-wave (cm/s)	12.7 ± 2.1	13.0 ± 1.6	0.439
FAC, %	45.7 ± 6.1	48.3 ± 7.5	0.082
IVCT, ms	69.8 ± 9.3	70.6 ± 8.9	0.663
IVRT, ms	66.0 ± 14.6	62.9 ± 11.3	0.288
TEI Index	0.51 ± 0.08	0.48 ± 0.07	0.178
RA area, cm^2^	15.3 ± 2.9	14.9 ± 2.3	0.422
RA volume, ml	46.7 ± 15.2	43.6 ± 10.3	0.289
RA volume Index, ml/m^2^	24.6 ± 7.6	23.1 ± 5.6	0.336
TV E wave velocity (cm/s)	59.7 ± 10.7	60.7 ± 11.7	0.703
TV A wave velocity (cm/s)	39.2 ± 7.8	36.7 ± 7.6	0.142
TV E/A	1.57 ± 0.35	1.69 ± 0.34	0.107
TV e′ wave velocity (cm/s)	12.7 ± 3.3	12.7 ± 2.3	0.930
TV a′ wave velocity (cm/s)	11.8 ± 4.2	9.8 ± 2.4	0.004
TV e′/a′ ratio	1.18 ± 0.48	1.371 ± 0.38	0.052
TV E/e′ ratio	4.93 ± 1.14	4.89 ± 1.13	0.872
TV E wave DT, ms	155.4 ± 30.4	165.6 ± 40.1	0.204
IVC diameter, cm	1.9 ± 0.3	1.9 ± 0.2	0.887
EPASP, mmHg	23.2 ± 4.0	22.5 ± 3.2	0.353

*DT* deceleration time, *EPASP* estimated pulmonary artery systolic pressure, *FAC* fractional area change, *IVC* inferior vena cava, *IVCT* isovolumetric contraction time, *IVRT* isovolumetric relaxation time, *RV* right ventricle, *TAPSE* tricuspid annular plane systolic excursion, *TV* tricuspid valve

The strain of all RV segments was significantly lower in smokers as compared to non-smokers, except for the free wall apical segment (Table 3). The basal RV free wall strain and basal septal wall strain were abnormally lower than the cut-off limit of − 20, based on the latest ASE guidelines [18]. The mean GLS of the RV was abnormal in chronic heavy cigarette smokers in contrast to the mean RV free wall strain which, although significantly lower in smokers than in non-smokers, was still within the currently accepted normal limits. The number of subjects with an abnormal RV GLS was significantly higher in the smokers’ group as compared to the normal control group (80% versus 8.6%, *p* < 0.001).

**Table 3:** Right ventricular strain of the 2 groups

Variable	Smokers (mean ± SD)	Non-smokers (mean ± SD)	***p*** value
Basal RV free wall strain, %	− 18.7 ± 6.2	− 27.2 ± 7.0	< 0.001
Mid RV free wall strain, %	− 20.6 ± 6.6	− 24.5 ± 6.3	0.007
Apical RV free wall strain, %	− 22.0 ± 6.6	− 24.8 ± 6.8	0.057
Basal septal strain, %	− 13.5 ± 4.2	− 19.5 ± 5.5	< 0.001
Mid septal strain, %	− 19.7 ± 4.6	− 25.8 ± 5.7	< 0.001
Apical septal strain, %	− 23.7 ± 5.8	− 27.9 ± 6.1	0.001
RV GLS, %	− 19.0 ± 3.2	− 24.5 ± 3.5	< 0.001
RV free wall strain, %	− 20.4 ± 4.1	− 25.5 ± 3.6	< 0.001

*RV* right ventricle, *GLS* global longitudinal strain

**Fig. 2:**
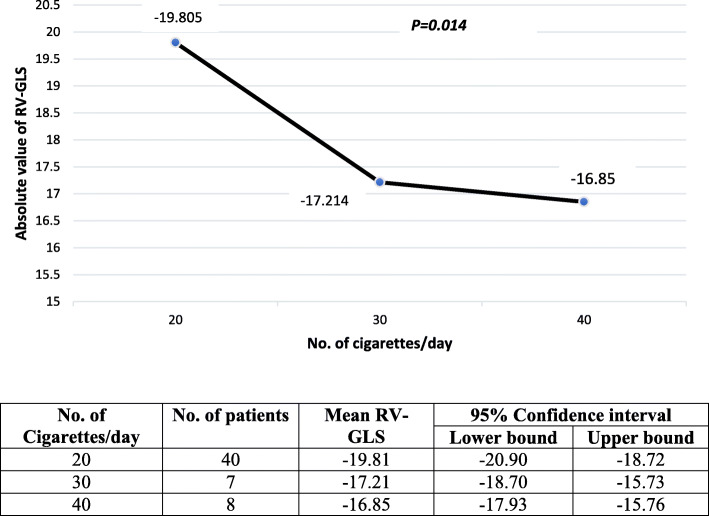
Means of RV GLS according to the number of consumed cigarettes per day

In the smokers group, the mean number of cigarettes smoked per day was 24.2 ± 7.4 cigarettes, the mean duration of smoking was 10.7 ± 5.9 years, and the mean consumption of cigarettes was 13.5 ± 10.4 pack-years. RV GLS was more adversely affected among those who smoked more cigarettes *per day* (Fig. 2). The impairment of RV GLS could not be correlated to the number of years of smoking (*r* = 0.140, *p* = 0.308) or the number of pack-years (*r* = 0.256, *p* = 0.057).

## Discussion

Cigarette smoking is one of the modifiable cardiovascular risk factors. Smoking has been linked to many cardiovascular diseases. In general, smokers have a higher risk of heart failure and poorer outcomes compared to never-smokers [19].

The relatively new technique of Speckle Tracking Echocardiography has revealed that chronic heavy smokers who develop COPD show echocardiographic evidence of right ventricular dysfunction appearing even before the occurrence of pulmonary hypertension and cor pulmonale. The present study aims to find out whether chronic cigarette smoking in otherwise healthy individuals can cause subtle subclinical dysfunction of the right ventricle by applying a 2-D speckle tracking echocardiography as well as other established echocardiographic parameters of right ventricular function.

Two groups of subjects were included, the first group included healthy non-cardiac individuals with history of chronic heavy cigarette smoking (*n* = 55), and the second group included healthy (never-smokers) subjects (*n* = 35).

In this study, the chronic heavy cigarette smokers had a higher pulse rate than non-smokers. Nicotine and PM2.5 are known to activate the sympathetic nervous system. Nicotine’s effect is acute, while PM2.5’s effect is on chronic basis. As shown in the study performed by Farsalinos et al. [20], the increase in pulse after smoking two cigarettes returned to baseline 30 min later. Thus, the main contributor to this higher pulse rate is most probably PM2.5 rather than nicotine. The acute effects on the autonomic nervous system were minimized as far as possible by requiring the included subjects not to smoke for at least 30 min before the echocardiographic study.

There was no significant difference in the blood pressure (office BP) between the two groups. Indeed, no chronic effects of smoking have been reported for office BP [21]. In the study by Farsalinos et al. [20] mentioned above, acute significant rise in office BP (by 8 mmHg systolic and 4 mmHg diastolic) after smoking two cigarettes returned to baseline 30 min later. Considering the fact that chronic heavy cigarette smokers rarely abstain from cigarette for more than 30 min at a stretch while awake, it would appear logical to expect that their blood pressure should be chronically raised. The fact that this was not the case in our study can be attributed to the use of office BP rather than ambulator blood pressure monitoring (ABPM), and the studied population abstained from smoking 30 min prior to their study. Studies using ABPM, in contrast to those using office BP, have shown that both normotensive smokers and untreated hypertensive smokers present higher daily BP values than their respective non-smoking counterparts [22].

The estimated pulmonary artery systolic pressure was not elevated in the smokers as well as in the non-smokers group (23.2 ± 4.0 mmHg and 22.5 ± 3.2 mmHg, respectively, *p* = 0.353). Keeping in mind that the echocardiographic assessment of the pulmonary artery systolic pressure has its limitations; nonetheless, there was no evidence that any of the subjects that participated in this study had pulmonary hypertension.

The conventional echocardiographic methods used for assessing the systolic and diastolic functions of the right ventricle showed normal measurements for both groups. Smokers had a lower FAC than non-smokers, but the difference was not statistically significant (*p* value = 0.082). Smokers had a higher TV a′ wave velocity and a lower TV e′/a′ ratio than non-smokers. These findings are in line with Eroglu et al. [23] who found no difference in the standard echocardiographic measurements of the right ventricle between a healthy group of 40 smokers and 40 non-smokers. In contrast, Ilgenli and Akpınar [13] found that cigarette smoking led to an impaired right ventricular diastolic function but not right ventricular systolic function in the acute period. This study is different from ours, since they investigated only the acute effects of smoking one cigarette on ventricular function and these effects were shown to disappear after 30 min.

In our current study, both groups were compared using the 2D-STE; healthy chronic heavy cigarette smokers had mild impairment in the mean GLS of the RV (mean value of − 19%), while non-smokers had a normal mean RV GLS (mean value of − 24.5%); this difference was statistically significant (*p* < 0.001). The RV segmental analysis showed a significant difference between both groups. The basal RV free wall strain (mean value of − 18.7) and basal septal strain (mean value of − 13.5) were the adversely affected segments (according to the currently accepted cut-off level) [16]. These two segments were mostly responsible for the mild impairment in the RV GLS in heavy chronic cigarette smokers.

This result is in line with the result of the study performed by Eroglu et al. [23] which showed that chronic cigarette smoking affects the systolic long-axis function of the right ventricle in healthy young subjects by strain imaging using Doppler myocardial velocity. In contrast to our study, Eroglu et al. also showed impairment of the diastolic function of the RV. In our study, despite the lower values of the RV diastolic function in smokers as compared to non-smokers, these values were still within the currently accepted normal limits. On the contrary, Ilgenli and Akpınar [13] found that cigarette smoking does not impair right ventricular systolic function in the acute period, but their study enrolled only 20 subjects, used tissue Doppler imaging as the method to calculate RV strain, and focused mainly on the acute effects of cigarette smoking.

Our study showed that the impairment in RV GLS correlated significantly to the number of cigarettes smoked per day, but could not be correlated to the number of years of smoking or the number of pack-years of smoking. However, it is well known that the association between smoking and cardiovascular diseases has been shown to be nonlinear, such that even a few cigarettes a day disproportionately increases cardiovascular risk [24]. One possible explanation for our finding is that large doses of the component/s of cigarette smoke that are responsible for causing RV dysfunction are required to cause saturation of the involved biochemical and cellular processes, thereby causing a linear dose-response relationship on RV function.

### Study limitations

As dedicated software for the speckle tracking of the right ventricle is not available; the software of the left ventricle was adapted to the right side. In theory, this should not have affected the study significantly as the same software was used for both smoking and non-smoking subjects. Furthermore, the region of interest was traced and manually adjusted to fit the right ventricle and the principles of speckle tracking remain unchanged irrespective of the site being studied. Another limitation of the study is that although all the subjects included in the study were asymptomatic, we could not exclude that some chronic heavy smokers had grade I COPD according to the latest gold criteria (GOLD grade I COPD is asymptomatic). This reasoning could be extended to include asymptomatic coronary artery disease as well as other diseases. Indeed, it would be impractical and beyond the scope of this study to carry out the required diagnostic tests to exclude all diseases. Moreover, the American College of Chest Physicians and the European Respiratory Society do not recommend screening of asymptomatic smokers by spirometry [25]. Indeed, the incidence of COPD in asymptomatic cigarette smokers is very low [26].

## Conclusion

The current study showed that chronic heavy cigarette smoking is associated with subtle subclinical dysfunction of the right ventricle which can be detected only by the more sensitive method of speckle tracking echocardiography, but not by the conventional echocardiography methods. Identification of these subtle changes can facilitate aggressive smoking cessation programs to encourage smokers to quit smoking before the development of clinical diseases. In order to determine whether the subtle smoking-induced dysfunction of the right ventricle is reversible or not, further studies are recommended.

## Data Availability

The datasets used and/or analyzed during the current study are available from the corresponding author on reasonable request.

## Abbreviations

2D: Two-dimensional
2D-STE: 2D-speckle tracking echocardiography
ABPM: Ambulator blood pressure monitoring
BMI: Body mass index
BP: Blood pressure
BSA: Body surface area
COPD: Chronic obstructive pulmonary disease
DBP: Diastolic blood pressure
EPASP: Estimated pulmonary artery systolic pressure
ET: Ejection time
FAC: Fractional area change
GATS: Global Adult Tobacco Survey
GLS: Global longitudinal strain
IVC: Inferior vena cava
IVCT: Isovolumetric contraction time
IVRT: Isovolumetric relaxation time
LV: Left ventricle
PASP: Pulmonary artery systolic pressure
ROI: Region of interest
RV: Right ventricle
SBP: Systolic blood pressure
SD: Standard deviation
TAPSE: Tricuspid annular plane systolic excursion
TDI: Tissue Doppler imaging
TV: Tricuspid valve
